# Local Hyperbaric Oxygen Therapy in the Treatment of Diabetic Foot Ulcers

**DOI:** 10.3390/ijerph191710548

**Published:** 2022-08-24

**Authors:** Jarosław Pasek, Sebastian Szajkowski, Piotr Oleś, Grzegorz Cieślar

**Affiliations:** 1 Faculty of Health Sciences, Jan Długosz University in Częstochowa, 13/15 Armii Krajowej St., 42-200 Częstochowa, Poland; 2 Department of Medical Sciences, Medical University of Mazovia in Warsaw, 8 Rydygiera St., 01-793 Warszawa, Poland; 3 Department of Internal Medicine, Angiology and Physical Medicine, Faculty of Medical Sciences in Zabrze, Medical University of Silesia in Katowice, 15 Stefana Batorego St., 41-902 Bytom, Poland

**Keywords:** computerized planimetry, treatment, physical medicine, local hyperbaric oxygen therapy, diabetic foot ulcers

## Abstract

**Background:** Diabetes mellitus is one of the most common metabolic diseases. The most serious complication of diabetes is diabetic foot ulcer, which affects several million people around the world each year. In recent years, increasingly modern methods of physical medicine including hyperbaric oxygen therapy have been used often in the complex therapy of this complication. **Methods:** This study included 45 patients, 24 male (53.3%) and 21 female (46.6%), whose age was between 49 and 83 years (mean age: 66.7 ± 8.8 years) with diabetes lasting for 1.5–18 years, who underwent local hyperbaric oxygen therapy at the pressure of 2.5 ATA (30 exposures for 30 min each) due to diabetic foot ulcers. The progress in wound healing before and after the end of therapy was evaluated by computerized planimetry, and the pain intensity was assessed with the use of a VAS. **Results**: The analysis of results showed a statistically significant reduction in the wound surface area after the treatment, from 8.54 ± 3.34 cm to 4.23 ± 3.23 cm² (*p* = 0.000001). In 5 patients (11.1%), the wounds were healed completely. In 25 patients (55.5%), the topical state of the wound surface was significantly decreased by 50% on average. There was also a significant reduction in the perceived pain on the VAS in all examined patients from 4.64 ± 1.68 points before treatment to 1.51 ± 0.92 points after treatment (*p* = 0.000001). **Conclusions:** The application of local HBO therapy in the treatment of diabetic foot ulcers accelerates the ulcer healing process, as judged in objective planimetric assessment, and reduces the intensity of perceived pain ailments.

## 1. Introduction

Diabetes mellitus is a chronic metabolic disease, one of the most serious complications of which is the diabetic foot ulcer (DFU) [1]. According to The International Working Group on Diabetic Foot (IWGDF), progressive changes in the foot may lead to the ulceration and/or destruction of deep tissues, in conjunction with neurological disorders and peripheral vascular diseases of varying degrees of severity [2]. The social nature of DFU is evidenced by the high frequency of its occurrence—this syndrome develops in about 15% of diabetic patients [3]. DFU manifests itself with troublesome, chronic pain; causes significant difficulties in movement; and, in its advanced stage, due to the fact that the ongoing pathological processes are progressive, may lead to tissue necrosis and limb amputation. According to the WHO, it is the sixth leading cause of death in the world [4]. In Poland, according to the data from the Central Statistical Office, as of 2016, the number of people suffering from DFU over 15 years of age was 2.1 million [5].

The problem of DFU is considered a serious challenge for modern medicine. A growing clinical problem is the still-large number of lower limb amputations performed in its course [6,7]. In order for this indicator to decrease, it is extremely important to take preventive measures, provide appropriate treatment, educate patients and make them aware of the need for a quick response in the event of disturbing symptoms. It should be remembered that in many cases, the mere conduct of a personal (interview) and physical (physical) examination allows for making the appropriate diagnosis [1,8,9].

Diabetic foot ulcers, as a complication of long-term hyperglycemia, arises from the coexistence of neuropathy and peripheral arterial disease (PAD). The damage caused to the sensory fibers leads to impaired perceptions of pain, temperature and vibration, the consequences of which are initially unnoticed injuries and micro-injuries to the feet in the form of wounds and the hardening of the epidermis. Disturbances in the functioning of the motor fibers lead to muscle atrophy and, as a result, to the deformation of the feet and an increased risk of injury. This triad of symptoms, neuropathy, foot deformity and slow-healing wounds, is present in approximately 60% of diabetic foot patients [10]. The risk factors leading to the occurrence of DFU include, among others: obesity, stimulants (smoking and excessive alcohol consumption) and neglect in foot care, i.e., lack of proper hygiene and use of improper footwear [11,12].

Local hyperbaric oxygen therapy (HBO) is a physical procedure involving the topical application of 100% oxygen or a mixture of high-oxygen gases at elevated pressure, and it is often used to treat poorly healing foot ulcers in patients with diabetes [13]. The currently available scientific evidence shows that there are statistically significant differences in favor of the use of HBO in the treatment of diabetic foot syndrome, compared with standard therapeutic management [14,15,16].

The beneficial therapeutic effect of hyperbaric oxygen therapy in diabetic foot ulcers is mainly related to the analgesic, anti-inflammatory (related to the reduction of the secretion of pro-inflammatory acute phase proteins, interleukins and cytokines), anti-swelling and regenerative effects leading to the acceleration of wound healing, the stimulation of angiogenesis (increased secretion of growth factor and pro-angiogenesis cytokines), the improvement of local blood flow and thermoregulation in tissues, and the delivery of an increased amount of oxygen to ischemic and hypoxic tissues, as well as bactericidal action and support of the mechanisms of immune system [13,15,17,18,19,20].

### Aim of the Study

In our opinion, confirmed by the authors of asystematic review of the literature data [21], the results of clinical trials to date are not sufficient for developing detailed guidelines on the use of HBO procedures in the treatment of diabetic foot syndrome, and there is a need for more studies that analyze the impacts of various factors on the therapeutic effect of this method.

That is why the aim of this study was to evaluate the effectiveness of treating diabetic foot ulcers with local hyperbaric oxygen therapy through a planimetric evaluation of the ulcers’ healing process and assessments of the intensity of accompanying pain, using a VAS, with regard to the gender and age of patients.

## 2. Material and Methods

The study was conducted in accordance with the decision of the local bioethical committee, issued by the Medical University of Silesia in Katowice, Poland (approval reference number: KNW/0022/KB1/102/II/16/19). Every patient enrolled in the study signed a written informed approval for all procedures conducted in the study. The protocol of the study has been designed according to the Declaration of Helsinki (1964).

The study involved 45 patients (24 men and 21 women) with long-term diabetes lasting from 1.5 to 18 years, age range from 49 to 83 years (mean: 66.7 ± 8.8 years), hospitalized in the Department of Internal Medicine, Angiology and Physical Medicine in Bytom, with the diagnosis of mixed diabetic foot ulcers (neuropathic-ischemic) in the period 2018–2020. No patient had undergone revascularization before participation in the study.

The inclusion criteria comprised: patient’s approval for participation in the trial, diabetic foot ulcer diagnosed in the foot area, age range between 40 and 85 years. The exclusion criteria included: lack of patient’s approval for participation in the trial, age beyond the range of 40–85 years, limb ulcer not associated with diabetic foot syndrome, acute ischemia of lower extremity, profound phlebothrombosis, infection that needed generalized antibiotic therapy and presence of contraindications to HBO therapy.

Before starting a cycle of local hyperbaric oxygen therapy procedures, if it was necessary, surgical wound debridement was conducted to remove necrotic tissues or purulent infiltration accompanying the ulcer.

The therapeutic effects of ulcer healing were assessed with the planimetric method, using an original computer program developed by Michał Senejko, MSc., that enabled the measurement of ulcer surface area in manual mode.

Following this method, on the picture of the ulcer obtained from a digital photo, the researcher moved a mouse cursor along the contour of the ulcer, with the subsequent automatic closure of the drawn contours and the creation of a closed curve rendering/expressing the precise shape and size of the ulcer. Afterwards, the program automatically calculated the ulcer surface area within the previously defined contour. The primary results of measurements were obtained in pixels, after the calibration of the distance of the ulcer surface from the camera lens and subsequently performing the scaling process and adequate calculations the results were presented in square centimeters, precisely describing the size of ulcer area.

Additionally, before and after the end of a cycle of local hyperbaric oxygen therapy procedures, the pain intensity assessment was conducted with the use of the VAS. 

The results of measurements of the ulcer surface area and pain intensity, conducted before the beginning of local hyperbaric oxygen therapy and after its completion for the whole group of patients were also compared in particular subgroups of patients with regard to their gender, age, and ulcer location.

### 2.1. Oxygen Hyperbaric Therapy Procedure

The patients were treated with the OXYBARIA-S device (FASSER S.A., Tarnowskie Góry, Poland) applied for procedures of local hyperbaric oxygen therapy [22].

The procedures were performed in semi-reclining position. The treated limb was placed in the therapeutic chamber, which was then closed at thigh level by means of a flexible sealing flange. The limb with the diabetic foot was subsequently subjected to physical treatment. The oxygen applied was introduced into the chamber from an external cylinder, and the oxygen concentration was about 95%, at the pressure of 2.5 ATA with the applied flow rate of 5 L/min. The performed procedures lasted for 30 min each and were applied once a day. A cycle of HBO therapy consisted of 30 procedures. The procedures were conducted in 2 series, comprising 15 daily procedures each (excluding Saturdays and Sundays). There was a 4-week interval between series of procedures.

During a cycle of physical procedures, topical pharmacological treatment was applied with the use of Allevyn Adhesive Ag dressing in order to maintain the required humidity and sterility of the wound.

### 2.2. Statistical Analysis

Statistical analysis was performed using Statistica 13 package (Statsoft, Kraków, Poland). The results are presented as means and standard deviation. The Wilcoxon signed-rank test was used to test the statistical significance of the differences in the examined parameters before and after the applied treatment. The significance of the differences between the individual subgroups was tested using Mann–Whitney and Kruskal–Wallis U tests. The level of statistical significance was set at *p* < 0.05.

## 3. Results

The mean values of ulcer surface area, as assessed using the planimetric method, before and after the end of a cycle of local hyperbaric therapy, for the whole group of patients and in particular subgroups singled out with regard to gender, age and ulcer location, with the results of statistical analysis, are included in Table 1.

Following a cycle of local hyperbaric oxygen therapy, a statistically significant decrease was observed (*p* = 0.000001) in mean ulcer area from 8.54 ± 3.34 cm^2^ before treatment to 4.23 ± 3.23 cm² after the treatment. Similarly, a statistically significant decrease in mean ulcer area was also achieved in particular subgroups: gender, male (*p* = 0.000018) and female (*p* = 0.000060); age, <60 years (*p* = 0.003346), 60–70 years (*p* = 0.000438) and >70 years (*p* = 0.000196); ulcer location, left foot vs. (*p* = 0.000008) and right foot (*p* = 0.000132).

The mean scores for pain intensity assessed on the VAS both before and after the end of a cycle of local hyperbaric therapy, for the whole group of patients and in particular subgroups singled out with regard to gender, age and ulcer location, with the results of statistical analysis, are included in Table 2.

As a result of a cycle of local hyperbaric oxygen therapy procedures, a statistically significant decrease was observed (*p* = 0.000001) in mean pain intensity from 4.64 ± 1.68 points before treatment to 1.51 ± 0.92 points after treatment. Similarly, a statistically significant decrease in mean pain intensity score was also obtained according to gender: male (*p* = 0.000003) and female (*p* = 0.000013); age: <60 years (*p* = 0.002569), 60–70 years (*p* = 0.000177) and >70 years (*p* = 0.000062); and ulcer location: left foot (*p* = 0.000001) and right foot (*p* = 0.000036).

Table 3 presents the means of the percentage changes in ulcer surface area assessed by planimetric method as well as the mean percentage changes in pain intensity assessed with the VAS after the completion of a cycle of local hyperbaric therapy according to gender, age, and ulcer location, with the results of statistical analysis.

There was no increase in the ulcer surface area in any of the patients after the treatment with local hyperbaric oxygen therapy. In 5 patients (11.1%), the wounds healed completely. Ulcer surface area reduction above 50% of the baseline was achieved in 25 patients (55.5%), and wound area decreased below 50% of the baseline value in 20 patients (44.4%). The mean percentage of ulcer area change for the entire group of patients was 55.0 ± 0.24%. Percentage changes in ulcer surface area after the end of treatment did not differ significantly between subgroups (Table 3).

In 5 patients (11.1%), complete pain relief was achieved. The least profound improvement in pain, i.e., 33.3%, was obtained in one patient. No pain intensification was found in any of the patients after treatment with local hyperbaric oxygen therapy. In 44 patients (97.7%), a reduction in pain intensity above 50% of the baseline was achieved, while only in 1 patient (2.2%), the reduction in pain intensity did not exceed 50% of the baseline values. The mean percentage change in pain intensity for the entire group of patients was 68.3 ± 0.15%. The percentage changes in pain intensity after the end of treatment did not differ statistically in particular subgroups (Table 3).

The results of measurements concerning the area of treated ulcers and the results according to VAS scale did not differ between the assessed groups, both at baseline and after the study.

## 4. Discussion

The treatment of diabetic foot ulcers requires a number of measures, primarily in the metabolic control of diabetes, the use of appropriate specialist dressings, the application of a proper diet, and appropriate footwear and orthopedic insoles, whether full-contact or those aimed at relieving the foot affected by the disease. Surgical treatment is also necessary in justified cases. The conservative methods used in the comprehensive therapy of diabetic foot syndrome include: vacuum VAC therapy, PRP method (dressings with the use of platelet-rich plasma), regular physical exercise and selected physical medicine procedures, including local hyperbaric oxygen therapy [8,23,24]. According to the latest standards for the treatment of DFU ulcers, the MOIST strategy should be followed: M—moisture—moist wound management, O—oxygen—care for oxygenation of the wound bed tissues, I—infection and inflammation control—control of microbial load and inflammation, S—support—supporting the healing processes, e.g., by relieving the foot, T—tissue debridement/management—cleaning the wound [25].

In the case of local HBO, increasing the partial pressure of oxygen that is applied directly to the tissues of the treated limb results in better oxygenation and increases the amount of oxygen that can be dissolved in the patient’s serum, according to Henry’s law. Chronic wound hypoxia is associated with poor or no healing. Daily HBO sessions help to provide the treated area with the right amount of oxygen, thus contributing to the progression of the healing process from the inflammatory phase to the proliferation phase. The formation of new blood vessels also depends on the correct level of tissue oxygenation. Hyperbaric oxygen therapy creates an oxygen concentration gradient between low oxygenated tissues in the center and better oxygenated tissues on the periphery, which provides the driving force favoring neovascularization [13,15,26].

In the presented study, after the application of local hyperbaric oxygen therapy, statistically significant improvement in the healing process was achieved, consisting of the reduction of the surface area of the treated ulcers.

Similar results were obtained by Glik et al., who assessed the effect of systemic hyperbaric oxygen therapy (HBOT) on ulcer healing in 142 patients with chronic venous insufficiency and diabetic foot syndrome who underwent a 30-day HBOT session; those authors verified the therapeutic effects using thermovision and computer planimetry. The obtained results showed a significant reduction in the area of the treated wounds after HBOT treatment in both groups of patients assessed [27].

Planimetric assessment was also used by Kawecki et al. to verify the therapeutic efficacy of HBOT in the treatment of 94 patients with vascular diabetic foot syndrome. In 26 patients, the ulcers healed completely, and in 37, the local condition of wounds improved significantly; the wound surface area decreased by an average of 34% [28].

Moreover, a systematic review and meta-analysis of 20 randomized and 1263 non-randomized clinical trials conducted by Zhang et al. confirmed that HBOT significantly increases the healing rate of diabetic foot ulcers (*p* < 0.001), shortens the healing time (*p* < 0.001) and reduces the incidence of major leg amputations (*p* < 0.01) [29].

Additionally, the meta-analysis of 11 randomized controlled trials (including 668 patients) comparing the effects of the standard treatment of diabetic foot ulcers and the standard treatment associated with HBOT published by Moreira et al. proved that patients treated with HBOT faced a lower risk of major leg amputations, had greater chances for ulcer healing and revealed higher percentage of ulcer surface area reduction after 2 weeks of HBOT [30].

A statistically significant reduction in the intensity of perceived pain ailments in the studied group of patients can also be considered a therapeutic success in the study presented here.

The analgesic effect of hyperbaric oxygen therapy reported in our study is confirmed by the literature analysis of MEDLINE, Embase and Cochrane Library carried out by Stoekenbroek et al. according to which this form of therapy is appropriate and brings measurable benefits not only in terms of accelerating the wound healing process and reducing the number of amputations performed but also reducing the intensity of perceived pain [31].

Another analysis carried out by Bishop and Mudge also confirmed the beneficial effect of HBO on the wound healing process and reduced risk of amputation, as well as a significant reduction in the intensity of perceived pain. In only one of the analyzed studies did the authors not find differences in the therapeutic results between patients receiving hyperbaric oxygen and those in the control group [32].

Based on the results of the presented study and the literature data, it may be assumed that in many cases, the use of modern physical methods including local hyperbaric oxygen therapy is important in the treatment of difficult-to-heal wounds, including ulcers occurring in the course of diabetic foot ulcer. Due to advances in technology and biomedical engineering, devices for physical therapy are becoming smaller, lighter, easier to use and cheaper, which increases their availability outside of specialized hospital wards. In addition, it is worth emphasizing the high safety profile of physical methods and the good tolerance of procedures carried out with their use by patients.

Despite the increasing number of publications appearing in the Polish and foreign literature on the beneficial effects of the clinical use of hyperbaric oxygen in the conservative treatment of diabetic foot, no clear guidelines have been established for practitioners in this regard so far [12]. In the analysis by Löndahl and Boulton of the literature on the effectiveness of HBO in the treatment of ischemic or neuro-ischemic ulcers of the lower extremities, the authors indicate ambiguous results regarding the treatment of DFU and emphasize the need for further multicenter studies on the use of HBO in the treatment of this disease, aimed at developing detailed guidelines concerning the use of HBO therapeutic procedures in the comprehensive treatment of diabetic foot syndrome [21].

The strength of the study is in the unquestionable confirmation of the therapeutic efficacy of local hyperbaric oxygen therapy in the treatment of diabetic foot ulcer in the form of stimulating the healing process, resulting in significant reductions in ulcer surface area and significant decreases in the intensity of accompanying pain according to patients’ gender and age.

The study has some limitations, such as:

1. Relatively small patient cohort,
2. Lack of information on the therapeutic procedures used previously,
3. Lack of a long-term follow-up,
4. Lack of a control group, which would have allowed for comparison with patients of similar characteristics treated with the use of routine methods, in order to eliminate other factors that could delay wound healing, such as type of wound, source of wound, duration of diabetes mellitus, average HbA1c level and medication prescribed (insulin or secretagogues and/or antibiotics).

Due to the presented limitations, this study could be considered a preliminary one.

## 5. Conclusions

The use of local hyperbaric oxygen therapy in patients with diabetic foot ulcer reduces the surface area of the treated wounds, assessed in objective planimetric assessment, and reduces the patients’ perceived pain.

## Figures and Tables

**Table 1:** The ulcer surface area (mean ± standard deviation SD) assessed with the planimetric method, before and after the end of local hyperbaric oxygen procedures, for the whole group of patients and by gender, age, and ulcer location.

		Ulcer Surface Area (cm²)		
		before Treatment	after Treatment	
	n (%)	Mean ± SD	Mean ± SD	*p*
Total	45 (100%)	8.54 ± 3.34	4.23 ± 3.23	0.000001
Gender				
male	24 (53.3%)	8.98 ± 3.67	4.62 ± 3.39	0.000018
female	21 (46.6%)	8.03 ± 2.94	3.79 ± 3.05	0.000060
*p*		0.372438	0.360390	
Age (in years)				
<60	11 (24.4%)	6.66 ± 2.37	2.79 ± 1.44	0.003346
60–70	16 (35.5%)	8.96 ± 3.58	4.51 ± 3.13	0.000438
>70	18 (40%)	9.31 ± 3.36	4.87 ± 3.9	0.000196
*p*		0.1411	0.4616	
Foot				
left	26 (57.7%)	8.23 ± 3.19	4.13 ± 3.38	0.000008
right	19 (42.2%)	8.95 ± 3.6	4.38 ± 3.09	0.000132
*p*		0.690505	0.758774	

**Table 2:** The intensity of pain assessed using the VAS, before and after the end of local hyperbaric oxygen therapy procedures, for the whole group of patients and by gender, age, and ulcer location.

		VAS Score (Points)		
		before Treatment	after Treatment	
	n (%)	Mean ± SD	Mean ± SD	*p*
Total	45 (100%)	4.64 ± 1.68	1.51 ± 0.92	0.000001
Gender				
male	24 (53.3%)	4.75 ± 1.64	1.54 ± 0.88	0.000003
female	21 (46.6%)	4.52 ± 1.74	1.47 ± 0.98	0.000013
*p*		0.660359	0.813044	
Age (in years)				
<60	11 (24.4%)	4.45 ± 1.96	1.63 ± 0.8	0.002569
60–70	16 (35.5%)	4.75 ± 1.57	1.5 ± 1.09	0.000177
>70	18 (40%)	4.66 ± 1.68	1.44 ± 0.85	0.000062
*p*		0.9658	0.8893	
Foot				
left	26 (57.7%)	4.5 ± 1.77	1.46 ± 0.9	0.000001
right	19 (42.2%)	4.84 ± 1.57	1.57 ± 0.96	0.000036
*p*		0.487223	0.707367	

**Table 3:** The mean percentage change of the ulcer surface area assessed by planimetric method and pain intensity assessed using a VAS after the applied treatment with local HBO by gender, age, and ulcer location, including the results of statistical analysis.

		(%) Change of Ulcer Surface Area after Treatment		(%) Change of VAS Score after Treatment	
	n (%)	Mean ± SD	*p*	Mean ± SD	*p*
Gender					
male	24 (53.3%)	−0.54 ± 0.23	0.7848	−0.67 ± 0.15	0.954
female	21 (46.6%)	−0.56 ± 0.26		−0.69 ± 0.15	
Age (in years)					
<60	11 (24.4%)	−0.56 ± 0.2	0.9326	−0.61 ± 0.11	0.208
60–70	16 (35.5%)	−0.53 ± 0.23		−0.72 ± 0.15	
>70	18 (40%)	−0.55 ± 0.28		−0.69 ± 0.16	
Foot					
left	26 (57.7%)	−0.54 ± 0.27	0.9358	−0.67 ± 0.16	0.809
right	19 (42.2%)	−0.55 ± 0.21		−0.69 ± 0.14	

## Data Availability

The datasets used and/or analyzed during the reported study are available from the corresponding author on reasonable request.

## Abbreviations

ATA	total atmosphere
VAS	visual analogue scale
HBO	hyperbaric oxygen therapy
DFU	diabetic foot ulcer
IWGDF	International Working Group on Diabetic Foot
WHO	World Health Organization
PAD	peripheral arterial disease
PRP	platelet rich plasma
VAC	vacuum therapy

## References

[1] Korzonek M., Markel A., Czarnota-Chlewicka J. (2016). Diabetic foot syndrome—Problem still valid. Pielęgn Chir Angiol..

[2] Lipsky B.A., Senneville É., Abbas Z.G., Aragón-Sánchez J., Diggle M., Embil J.M., Kono S., Lavery L.A., Malone M., van Asten S.A. (2020). Guidelines on the diagnosis and treatment of foot infection in persons with diabetes (IWGDF 2019 update). Diabetes Metab. Res. Rev..

[3] Drela E., Mielcarz G. (2017). Ischemic diabetic foot syndrome—Form epidemiology to diagnostics. Pielęgn Chir Angiolog..

[4] Central Statistical Office (2018). Infografika World Diabetes Day.

[5] Topor-Madry R., Wojtyniak B., Strojek K., Rutkowski D., Bogusławski S., Ignaszewska-Wyrzykowska A., Jarosz-Chobot P., Czech M., Kozierkiewicz A., Chlebus K. (2019). Prevalence of diabetes in Poland: A combined analysis of national databases. Diabet Med..

[6] Sun J.H., Tsai J.S., Huang C.H., Lin C.H., Yang H.M., Chan Y.S., Hsieh S.H., Hsu B.R., Huang Y.Y. (2012). Risk factors for lower extremity amputation in diabetic foot disease categorized by Wagner classification. Diabetes Res. Clin. Pract..

[7] Czeleko T., Śliwczyński A., Nawrot I., Karnafel W. (2014). The incidence of major non-traumatic lower limb amputations in people without diabetes in Poland in 2009–2012 based on the database of the National Health Fund. Acta Angiol..

[8] Clinical Recommendations for the Management of Diabetes Mellitus (2020). The position of the Polish Diabetes Society. Clin. Diabetol..

[9] Mieczkowski M., Siwko T.J., Parafiniuk J., Mrozikiewicz-Rakowska B., Krasnodębski P., Krzymień J., Czupryniak L. (2015). Health behaviors of diabetic patients in the prevention of diabetic foot syndrome. Wound Treat..

[10] Nehring P., Mrozikiewicz-Rakowska B., Krzyżewska M., Sobczyk-Kopcioł A., Płoski R., Broda G., Karnafel W. (2014). Diabetic foot risk factors in type 2 diabetes patients: A cross-sectional case control study. J. Diabetes Metab. Disord..

[11] Bahktiani P., Mansuri O., Yadav A., Osuoha C., Knight P., Baynosa R., McLafferty R., Jakoby M. (2015). Impact of hyperbaric oxygen on diabetic ulcers is unaffected by glycaemic control. Undersea Hyperb. Med..

[12] Lipiński P. (2021). Rules for the treatment of diabetic foot ulcer infection on the basis of current guidelines and reports. Wound Treat..

[13] Sieroń A., Cieślar G., Kawecki M. (2006). Outline of Hyperbaric Medicine.

[14] Liu R., Li L., Yang M., Boden G., Yang G. (2013). Systematic review of the effectiveness of hyperbaric oxygenation therapy in the management of chronic diabetic foot ulcers. Mayo Clin. Proc..

[15] Piotrowska A., Zych M., Oliwa J. (2021). Application of Hyperbaric Oxygen Therapy in the skin diseases treatment. Med. Rehabil..

[16] Walewska E., Ścisło L., Puto G., Klich M., Szczepanik A. (2016). The use of hyperbaric oxygen in the treatment of diabetic foot syndrome—Own experience. Wound Treat..

[17] Kirby J.P. (2019). Hyperbaric oxygen indications: Diabetic foot ulcers and intractable management. Mo. Med..

[18] Englisz B., Cholewka A., Firganek E., Knefel G., Liszka G., Kawecki M., Nowak M., Sieroń K., Stanek A. (2020). Evaluation of hyperbaric oxygen therapy effects in hard-to-heal wounds studied by thermal imaging and planimetry. J. Therm. Anal. Calorim..

[19] Kasprzyk-Kucewicz T., Cholewka A., Englisz-Jurgielewicz B., Mucha R., Relich R., Kawecki M., Sieroń K., Onak P., Stanek A. (2021). Thermal effects of topical hyperbaric oxygen therapy in hard-to-heal wounds—A pilot study. Int. J. Environ. Res. Public Health.

[20] De Wolde S.D., Hulskes R.H., Weenink R.P., Hollmann M.W., Van Hulst R.A. (2021). The effects of hyperbaric oxygenation on oxidative stress, inflammation and angiogenesis. Biomolecules.

[21] Löndahl M., Boulton A.J.M. (2020). Hyperbaric oxygen therapy in diabetic foot ulceration: Useless or useful? A battle. Diabetes Metab. Res. Rev..

[22] Pasek J., Sieroń A. (2015). OXYBARIA-S—An innovative device for hyperbaric oxygen therapy. Pract. Rehabil..

[23] Kaiva K., Loupa C., Vasilopoulos G., Govina U., Kalemikerakis I. (2021). Care of diabetic foot ulcers with hyperbaric oxygen therapy. Int. J. Lower Extr. Wounds.

[24] Gębala-Prajsnar K., Stanek A., Pasek J., Prajsnar G., Berszakiewicz A., Sieroń A., Cholewka A. (2015). Selected physical medicine interventions in the treatment of diabetic foot syndrome. Acta Angiol..

[25] Nuutila K., Eriksson E. (2021). Moist Wound Healing with Commonly Available Dressings. Adv. Wound Care.

[26] Health Quality Ontario (2017). Hyperbaric Oxygen Therapy for the treatment of diabetic foot ulcers: A health technology assessment. Ont. Health Technol. Assess. Ser..

[27] Glik J., Cholewka A., Stanek A., Englisz B., Sieroń K., Mikuś-Zagórska K., Knefel G., Nowak M., Kawecki M. (2019). Thermal imaging and planimetry evaluation of the results of chronic wounds treatment with hyperbaric oxygen therapy. Adv. Clin. Exp. Med..

[28] Kawecki M., Pasek J., Cieślar G., Sieroń A., Knefel G., Nowak M., Glik J. (2018). Computerized planimetry evaluation of hyperbaric oxygen therapy in the treatment of diabetic foot. Adv. Clin. Exp. Med..

[29] Zhang Z., Zhang W., Xu Y., Liu D. (2022). Efficacy of hyperbaric oxygen therapy for diabetic foot ulcers: An updated systematic review and meta-analysis. Asian J. Surg..

[30] Moreira D.A., Cruz D.L., Oliveira-Pinto J., Mansilha A. (2022). The role of hyperbaric oxygen therapy in the treatment of diabetic foot ulcers: A systematic review with meta-analysis of randomized controlled trials on limb amputation and ulcer healing. Int. Angiol..

[31] Stoekenbroek R.M., Santema T.B., Legemate D.A., Ubbink D.T., van den Brink A., Koelemay M.J. (2014). Hyperbaric oxygen for the treatment of diabetic foot ulcers: A systematic review. Eur. J. Vasc. Endovasc. Surg..

[32] Bishop A.J., Mudge E. (2014). Diabetic foot ulcers treated with hyperbaric oxygen therapy: A review of the literature. Int. Wound J..

